# The Influence of Gender on Food Consumption Patterns Among National-Level Adolescent Cyclists

**DOI:** 10.7759/cureus.33576

**Published:** 2023-01-09

**Authors:** Vani B Golla, Raj Kapoor, Khushi Khandelwal, Tamoghna Ghosh, Kanwal P Kochhar

**Affiliations:** 1 Department of Physiology, Indraprastha University, New Delhi, IND; 2 Department of Physiology, Vardhman Mahavir Medical College and Safdarjung Hospital, New Delhi, IND; 3 Department of Physiology, All India Institute of Medical Sciences, New Delhi, New Delhi, IND; 4 Department of Medicine, All India Institute of Medical Sciences, New Delhi, New Delhi, IND

**Keywords:** food habit, eating behaviour, cyclist, athlete, adolescent

## Abstract

Background and objective

Adolescents in general make poor food choices due to a lack of awareness, social pressure, and other factors, leading to a faulty lifestyle. On the other hand, the adolescent athletic population is associated with a healthy eating pattern. In light of this, this study aimed to evaluate the eating behavior of adolescent cyclists competing at the national level.

Methods

A total of 50 national-level adolescent cyclists (26 males and 24 females) were assessed for eating behavior, daily food consumption patterns, and eating habits around exercise time by using a pre-tested validated questionnaire, Food Frequency Questionnaire (FFQ), and 24-hour Food Recall.

Results

The majority (82%) of the cyclists were non-vegetarians, followed by lacto-vegetarians (14%) and lacto-ovo vegetarians (14%). Of note, 72% of the cyclists consumed four meals consisting of breakfast, lunch, snacks, and dinner daily, while 28% skipped at least one of the meals. The preference for takeaways (52%) surpassed dine-outs (34%) and home-cooked (14%) food. Pre-training snack was consumed by 37% and post-training snack by 47%. Cyclists daily consumed breakfast cereals (76%), bread (94%), pulses (92%), fruits (100%), vegetables (62%), milk (84%), milk products (90%), egg (82%), poultry, fish, and meat (74%), dry fruits, nuts, and seeds (78%), and saturated fats (100%). Junk foods (94%) and sweetened beverages (70%) were consumed at least once a week. No significant difference was observed in eating behavior and daily food consumption pattern between male and female cyclists.

Conclusion

The eating behavior of adolescent cyclists was inclined towards the consumption of saturated fats, junk food, and sweetened beverages. Male and female athletes have similar food habits. There is a need for the implementation of behavior change-oriented nutrition strategies to inculcate healthy eating habits among adolescent cyclists.

## Introduction

Proper nutrition, a balanced diet, and regular physical activity have a beneficial effect on the growth and development of young athletes, promoting their endurance and health, which is particularly important during the period of adolescence. However, the availability of food rich in fat and carbohydrates has altered food preferences and dietary patterns, leading to the prevalence of an unbalanced diet among adolescents [1]. Poor dietary choices reduce the exercise capacity of athletes, and eating behavior is conditioned by environmental and individual factors.

Nutrition and eating habits have always received great attention in sports, especially given their effects on athletic performance [2]. Adolescent athletes’ concurrent sporting pursuits, training/competition, unhealthy eating environments during travel and at their training and competition locations add to their nutrition challenges, such as meeting nutrient needs for growth, maintenance of health, challenging schedules (school, training, socializing, work, etc.), lack of knowledge, and reliance on others for the purchase and preparation of foods [3]. Although knowledge about healthy and safe food is certainly a precondition for a nutritious diet, the motivation to adopt the recommendations is also necessary [4]. Eating habits are acquired from the family since the earliest childhood, and then they are additionally influenced by the social and physical environment and the macro-system [5]. Consolidation of nutrition behavior and habits occurs by the age of 15 years with minimal changes between the ages of 15 and 18 years, i.e., between the middle (13-17 years) and late adolescence (17-19 years) [6,7].

Despite the prominent role that diet has on health and performance, athletes may not always make appropriate food choices [8]. Very few studies have explored individual and interpersonal determinants of food choices among athletes. Though there are reported similarities to the general population, there appear to be factors related to diet that are specific to athletes and distinctly related to performance [9]. Dietary intake in young athletes is higher than in their non-athletic counterparts [10]. One might think that athletes would be more conscious about their eating habits, which might differ from the general population. The aim of this research was to study eating behavior, food consumption patterns around exercise time, and the role of gender in food consumption patterns among national-level adolescent cyclists.

## Materials and methods

Research design and sampling

In this study, an exploratory study design with purposive sampling was employed. Adolescent cyclists inducted by the Cycling Federation of India (CFI) into the National Centre of Excellence (NCoE) of the Sports Authority of India based on CFI 2021 selection trials were recruited for the study. The study was carried out in August-September 2021. A total of 50 adolescents of both genders fulfilled the inclusion-exclusion criteria. All cyclists aged 14-19 years, training for a minimum of two years, practicing at least 15 hours per week, and had participated in either national- or international-level competitions were included in the study. Cyclists having any pathological conditions such as fever, hormonal imbalances such as hypothyroidism, polycystic ovarian syndrome (PCOS), and those in the rest phase of training were excluded. The authors retained the inclusion-exclusion criteria of a larger study on the same population. Written voluntary assent and informed consent to participate were obtained from each participant and the parent/guardian before participation in the study. The participants were free to withdraw from the study at any time without citing any reason. Ethical clearance was obtained from the Ethics Committee of the Sports Authority of India (ID: SAI/EC/2019-20/02/03).

Research tools

A questionnaire was designed incorporating questions broadly covering sociodemographics, food preferences, eating habits, and dietary patterns around training sessions. The questionnaire was validated for content and design by three nutritionists, tested on 10 athletes, internal consistency was studied, and all questions with a Cronbach α score of >0.7 were finalized. None of these participants was involved in the subsequent study.

Demographics and participant characteristics including food preferences, eating habits, and dietary patterns around training sessions were assessed using the pre-tested validated questionnaire, Food Frequency Questionnaire (FFQ), and 24-hour Food Recall for three training and one non-training day. Questions on eating behavior covered food preference, meals skipped and reasons for skipping, and habits of consuming food from joints/restaurants/street food as well as convenience foods. Daily food consumption and training meal behavior were studied based on 24-hour Food Recall and FFQ. The detailed questionnaire is provided in Appendices as supplementary data.

Sociodemographic Parameters

A family consisting of parents and their children was considered a nuclear family. A family that consisted of parents and their children, aunts, uncles, grandparents, and cousins all living in the same household was considered. an extended family. A family consisting of three or more generations of individuals and their spouses living together as a single household was considered a joint family.

Statistical analysis

Mean, standard deviation (SD), percentages, and frequencies were used for presenting participant characteristics and eating habits of cyclists. An independent t-test was performed to compare the means of food quantity consumed with respect to gender. The chi-square test of independence was performed to determine the association of gender with demographics and food habits. For observations in individual cells of cross-tabulation less than 10, Fischer’s exact test was used, while the Chi-square test was used for observations in individual cells greater than 10. A statistical probability level of p<0.05 (5%) was considered significant.

## Results

A total number of 50 cyclists participated in the study (26 males and 24 females). Of the total respondents, 52% were male. All the cyclists were between 14 and 19 years of age. The mean training years was 3.1 ± 1.2 (male: 3.7 ± 1.1; female: 2.6 ± 1.1) and the mean body mass index was 22.7 ± 1.8 (male: 33.9 ± 1.8; female: 22.6 ± 1.9). The demographic details are presented in Table 1.

**Table 1:** Gender-wise distribution of the demographic characteristics of cyclists NS: not significant; S: significant

Parameters	Male	Female	Total	P-value
	N (%)	N (%)	N (%)	
Age group (years)				
14-15	8 (30.8)	5 (20.8)	13 (26.0)	0.28^NS^
16-17	15 (57.7)	12 (50.0)	27 (54.0)	
18-19	3 (11.5)	7 (29.2)	10 (20.0)	
Type of family				
Nuclear	14 (53.8)	20 (83.3)	34 (68.0)	0.04^S^
Extended	8 (30.8)	4 (16.7)	12 (24.0)	
Joint	4 (15.4)	0	4 (8.0)	
Level of education				
High school	16 (61.5)	14 (58.3)	30 (60.0)	0.91^NS^
Senior secondary	9 (34.6)	8 (33.3)	17 (34.0)	
College	1 (3.8)	2 (8.3)	3 (6.0)	
Highest achievement in sports				
International medallist	5 (19.2)	1 (4.2)	6 (12.0)	0.21^NS^
International participation	3 (11.5)	2 (8.3)	5 (10.0)	
National medallist	13 (50.0)	11 (45.8)	24 (48.0)	
National participation	5 (19.2)	10 (41.7)	15 (30.0)	

The majority (80%) of the cyclists were in the phase of middle adolescence. Of note, 83% of the female cyclists belonged to nuclear families. The cyclists competed in age-group competitions organized by CFI. Male cyclists showed greater excellence in terms of medals won and participation in international competitions compared to their female counterparts. However, no significant difference was found between genders in terms of performance. Nearly half (48%) of the participants were national medallists in various age-group competitions. Out of 28 states and eight Union Territories (UTs) in India, the cyclists represented 15 and three respectively. The number of cyclists from each state/UT was predominantly between one and three, except Karnataka, which contributed the largest number of cyclists (seven), Manipur (six), and Haryana and Maharashtra (five each). The three Northern UTs include Chandigarh, Delhi, and Ladakh. Chandigarh, Ladakh, Odisha, Tamil Nadu, Uttarakhand, and West Bengal had only female participation while Andhra Pradesh, Delhi, and Rajasthan lacked any female participation. Manipur was the one state that had an equal distribution of male and female cyclists (Table 2).

**Table 2:** Nativity of national-level adolescent cyclists UT: Union Territory

State/UT	Male	Female	Total
	N (%)	N (%)	N (%)
Andhra Pradesh	1 (3.8)	0	1 (2.0)
Andaman and Nicobar Islands	2 (7.7)	1 (4.2)	3 (6.0)
Assam	1 (3.8)	2 (8.3)	3 (6.0)
Chandigarh	0	1 (4.2)	1 (2.0)
Delhi	2 (7.7)	0	2 (4.0)
Haryana	2 (7.7)	3 (12.5)	5 (10.0)
Karnataka	5 (19.2)	2 (8.3)	7 (14.0)
Kerala	2 (7.7)	0	2 (4.0)
Ladakh	0	2 (8.3)	2 (4.0)
Maharashtra	3 (11.5)	2 (8.3)	5 (10.0)
Manipur	3 (11.5)	3 (12.5)	6 (12.0)
Odisha	0	1 (4.2)	1 (2.0)
Punjab	1 (3.8)	1 (4.2)	2 (4.0)
Rajasthan	3 (11.5)	0	3 (6.0)
Tamil Nadu	0	3 (12.5)	3 (6.0)
Telangana	1 (3.8)	1 (4.2)	2 (4.0)
Uttarakhand	0	1 (4.2)	1 (2.0)
West Bengal	0	1 (4.2)	1 (2.0)

None of the cyclists were complete vegans or peso-vegetarians. Lactose intolerance (3.8%) and gluten allergy (7.7%) were observed in male cyclists. Lacto-vegetarians and lacto-ovo-vegetarians in the cohort belonged to Maharashtra, Uttarakhand, Odisha, Haryana, and Rajasthan. Of the total participants, 72% consumed four meals consisting of breakfast, lunch, mid-evening snacks, and dinner, while 28% skipped at least one of the four meals. Of the 14 cyclists who skipped meals, the reasons reported for skipping meals included feeling sleepy/drowsy and procrastination (42.9%), time constraint (28.6%), poor appetite (14.3%), and heavy preceding meals (14.3%). Intermittent snacking was observed in all cyclists who skipped meals. Of the 36 cyclists who did not skip any of the four meals, 19 (52.8%) engaged in intermittent snacking. The eating behavior of the participants is presented in Table 3.

**Table 3:** Eating behavior of adolescent cyclists NS: not significant; S: significant

Parameters	Male	Female	Total	P-value
	N (%)	N (%)	N (%)	
Food preference				
Lacto-vegetarian	4 (15.4)	3 (12.5)	7 (14.0)	0.99^NS^
Lacto-ovo-vegetarian	1 (3.8)	1 (4.2)	2 (4.0)	
Non-vegetarian	21 (80.8)	20 (83.3)	41 (82.0)	
Number of meals consumed				
3	7 (26.9)	7 (29.2.8)	14 (28.0)	0.86^NS^
4	19 (73.1)	17 (70.8)	36 (72.0)	
Meals skipped				
Breakfast	0	1 (4.2)	1 (2.0)	0.93^NS^
Lunch	6 (23.1)	6 (25.0)	12 (24.0)	
Dinner	1 (3.8)	0	1 (2.0)	
Don’t skip	19 (73.1)	17 (70.8)	36 (72.0)	
Place preference				
Home-cooked	5 (19.2)	2 (8.3)	7 (14.0)	0.46^NS^
Dine-out	7 (26.9)	10 (41.7)	17 (34.0)	
Takeaways	14 (53.8)	12 (50.0)	26 (52.0)	
Training food habits				
Pre-training	19 (73.1)	18 (75.0)	37 (74.0)	0.43^NS^
During training	20 (76.9)	16 (66.7)	36 (72.0%)	0.43^NS^
Post-training	26 (100)	21 (87.5)	47 (94.0)	0.06^NS^

In terms of the place preference for food, takeaways were the most popular (52%), followed by eating in restaurants/joints/food hubs/street food (34%), while the least (14%) preference was for the consumption of home-cooked food. Cyclists trained in two sessions: morning and evening (60-120 minutes). Foods consumed within 30 minutes from the start of a training session were considered "pre-training", food or fluids consumed intermittently during the training session were considered "during training", and food or fluid consumed within 30 minutes from the end of a training session was considered "post-training". Cyclists consumed biscuits (92%), dry fruits, nuts, and seeds (64.0%), bananas (58%), other fruits (32%), coffee (52%), and milk tea (48%) before training sessions. Fluids in the form of plain water, sports drink, or lemonade were consumed during training on track sessions while easy-to-eat convenience foods and fluids such as bananas (90%), bread-jam (80%), biscuits (58%), sports bars (28%), lemonade or sports drink (86%), and plain water (74%) were consumed on road-cycling days. Post-training eating patterns included the consumption of whey protein (94%) and fruit juice/smoothie (90%). The daily food consumption behavior of cyclists is summarized in Table 4.

**Table 4:** Daily food consumption behavior of cyclists *Weekly consumption NS: not significant; S: significant

Food groups	Male	Female	Total	P-value
	N (%)	N (%)	N (%)	
Breakfast cereals	19 (73.1)	19 (79.2)	38 (76.0)	0.62^NS^
Bread	25 (96.2)	22 (91.7)	47 (94.0)	0.51^NS^
Pulses	25 (96.2)	21 (87.5)	46 (92.0)	0.27^NS^
Fruit	26 (100)	24 (100)	50 (100)	0.32^NS^
Vegetables	17 (65.4)	14 (58.3)	31 (62.0)	0.62^NS^
Milk	25 (96.2)	17 (70.8)	42 (84.0)	0.01 ^S^
Milk products	25 (96.2)	20 (45)	45 (90.0)	0.14^NS^
Egg	22 (84.6)	19 (79.2)	41 (82.0)	0.62^NS^
Poultry, fish, and meat	17 (65.4)	20 (83.3)	37 (74.0)	0.15^NS^
Dry fruits, nuts, and seeds	22 (84.6)	17 (70.8)	39 (78.0)	0.25^NS^
Saturated fats	26 (100)	24 (100)	50 (100)	0.29^NS^
Junk food*	23 (88.5)	24 (100)	47 (94.0)	0.09^NS^
Sweetened beverages*	19 (73.1)	16 (66.7)	35 (70.0)	0.63^NS^

All cyclists consumed either rice or roti or both during lunch and dinner throughout the study period, as rice and/or roti is the staple food of Indians during lunch and dinner. Breakfast cereals - cornflakes, muesli, and oat flakes - were consumed by 76% while bread was consumed by 94% in the form of toast, bread-jam, bread-peanut butter, or bread-omelette. Pulses were consumed by 92% of cyclists in the form of sprouts or dhal preparations, with sprouts being the most popular choice for consuming pulses. Fruits in the form of whole fruit or juices were consumed by all cyclists (100%) with a marked preference for fresh fruit juices (90%) over whole fruit (76%). Among all the food groups, vegetable consumption was observed to be the least (62%), with a preference for raw vegetables or boiled vegetables over sabzi. Milk was the preferred choice among male cyclists compared to female cyclists (p<0.01). Eggs were consumed by 82% of cyclists while non-vegetarian foods such as chicken, fish, and meat were consumed by 74%. It was observed that eggs were consumed as a substitute for non-vegetarian foods due to certain customs and beliefs. This habit was prevalent among male cyclists. Saturated fats in the form of ghee, butter, or cheese were consumed by all cyclists. High-calorie foods consumed during dine-outs and as takeaways included burgers, and chicken in the form of fried chicken or biryani, and cyclists consumed them mostly on non-training days. The inclination towards junk food is presented in Table 5.

**Table 5:** Inclination of cyclists towards junk food

Convenience/junk food	Male	Female	Total
	N (%)	N (%)	N (%)
Dessert	1 (3.8)	0	1 (2.0)
Pizza	1 (3.8)	7 (29.2)	8 (16.0)
Momo	1 (3.8)	4 (16.7)	5 (10.0)
Burger	8 (30.8)	8 (33.3)	12 (24.0)
Noodles	3 (11.5)	2 (8.3)	5 (10.0)

## Discussion

Eating behavior governs several aspects of health, which, in turn, is affected by lifestyle parameters. In athletes, this is influenced by various stimuli present in their environment, such as those pertaining to coaches, parents, peers, and game-related stress [11]. Attitude plays a pertinent role in adopting and maintaining diverse health and nutritional habits. Socioeconomic factors also affect food selection and food intake in human societies [12]. Proper nutrition promotes the achievement of optimum athletic performance. In adolescent athletes, in addition to the energy requirements arising from exercise, appropriate dietary habits picked up in adolescence will be carried on to adulthood and, in parallel, thanks to their physical activity, the risk of incorrect lifestyle can be reduced [13]. Nutrition practices of elite athletes are reportedly subnormal both globally and among Indian athletes [14,15].

Breakfast and snacking habits impact total energy intake [16]. Skipping breakfast was observed among state-, national-, university-, and elite-level Indian athletes, while dinner was skipped by district-level Indian athletes. In contrast with the previous studies on Indian athletes [15,17-21], in our study, lunch was the most skipped meal (24%), the major reason being procrastination/drowsiness. Skipping meals may trap the athlete in a vicious cycle of tiredness and poor athletic performance, leading to psychological pressure and stress. The frequency of skipping ranged between three and seven days per week.

Physical activity and appropriate intakes of fruit and vegetables have a beneficial effect on bone-mineral accrual during childhood and adolescence [22]. Fruit consumption in Indian collegiate- and district-level athletes [15,21] and vegetable consumption in Indian collegiate athletes, volleyball players, weightlifters, runners, and male professional basketball players was low compared to the Recommended Dietary Allowance of Indians [15,19-21]. In our study, all athletes consumed fruit daily in the form of whole fruit or cut fruits and fruit juices. The consumption of almonds by Indian competitive wrestlers, as well as the consumption of dates (40 g) and cashew (50 g) by male professional basketball players of Dharwad city per day has been described [23]. In our study, dry fruits, nuts, and seeds were consumed by 78% of cyclists as "pre-", "during training" snacks, or as intermittent snacks (throughout the day).

Athletes usually consume meals rich in saturated fat [24]. The cyclists in this study consumed saturated fat daily in the form of butter. Fat consumption, particularly ghee, was high in Indian competitive wrestlers [23]. High consumption of junk foods (noodles, aloo chips, samosa, bread pakora, etc.) was common among state-/national-level sportswomen [22]. Complex carbohydrates are replaced by refined carbohydrates due to choices such as bread and processed snacks [25]. Compared to women, men, to a large extent, did not include vegetable fats and did not limit the consumption of animal fats, sweetened carbonated beverages, energy drinks, or fast-food products. Athletes training in individual sports disciplines consume hydrating beverages less often compared to those training in team sports [26].

The use of supplements is common among athletes. Multivitamin-mineral, vitamin C, vitamin D, sports bars, and protein powders were the most commonly consumed supplements on a regular basis [27]. The cyclists in this study consumed whey protein after every training session, sports bars during road cycling, and multivitamin-mineral every training day. Data related to the eating habits of adolescents showed that the intake of fruit, vegetables, milk, and dairy products should increase, while the intake of sweets, soft drinks, and food rich in fat should decrease. In addition, more frequent meals and regular breakfast consumption should be promoted, which is vital for more qualitative nutritional intake [28,29]. The scale of incorrect nutrition choices among athletes varies with gender, level of competitive participation, and type of sport practiced, with inappropriate behaviors more often seen in males than females and athletes competing at lower levels (non-master class). The nature of the performing discipline was a factor that did not much influence the nutritional choices of athletes [20]. In this study, no significant gender differences were observed in the eating behavior and food choices of cyclists.

This study has a few limitations, primarily its small sample size. All the cyclists were residents of the training facility of the Sports Authority of India and the results may not apply to cyclists living at home or other facilities as socioeconomic factors and parental influence could play an important role in influencing eating behavior.

## Conclusions

Based on our findings, the eating behavior of adolescent cyclists is inappropriate with a marked inclination towards consumption of saturated fats, junk food, and sweetened beverages, which is similar to the trends observed in the non-athletic adolescent population. No significant gender differences were observed among the cyclists in eating behavior and food selection. Attempts should be made to raise awareness about adopting and maintaining healthier dietary practices for improved performance. Nutritional education must be incorporated at various levels to promote good health. For a better understanding of the association between food habits and athletic performance, we recommend further studies with a larger study population, which should also include a questionnaire to analyze cognitive and stress scales.

## Appendices

The questionnaire/interview schedule for eliciting information from swimmers and cyclists is presented in Table 6.

**Table 6:** Questionnaire/interview schedule for eliciting information from swimmers and cyclists

Serial no.	Question	Options
1	Name	-
2	Date of birth	-
3	Gender	Male/female
4	Education	Middle school/high school/higher secondary school/undergraduate
5	Family profile	Joint/extended/nuclear
6	Native state	-
7	State representing in competitions	-
8	Highest rank/achievement in sports	-
9	How many years of training?	-
10	How many times do you train in a day?	Once/twice/more than twice
11	How many hours do you spend in a week?	<10, 10-11, 12-13, >13
12	What is your food preference?	-
13	How many meals do you consume in a day?	3/4/5/>5
14	Which meal do you skip often?	Breakfast/lunch/dinner/snacks
15	Reason for skipping meals?	-
16	Do you eat at joints/food outlets?	Yes/no
17	What will you choose?	dine-out/takeaway
18	What is your frequency of eating out?	Once in 2-3 months/once a month/once a fortnight/once a week/more than once a week
19	Do you suffer from any known/identified food-related problems?	Gluten sensitivity/lactose intolerance/any other/none
20	Record of days’ food and fluid intake consumed in the past 24 hours	-
